# Dengue Type 4 Live-Attenuated Vaccine Viruses Passaged in Vero Cells Affect Genetic Stability and Dengue-Induced Hemorrhaging in Mice

**DOI:** 10.1371/journal.pone.0025800

**Published:** 2011-10-28

**Authors:** Hsiang-Chi Lee, Yu-Ting Yen, Wen-Yu Chen, Betty A. Wu-Hsieh, Suh-Chin Wu

**Affiliations:** 1 Institute of Biotechnology, Department of Life Science, National Tsing Hua University, Hsinchu, Taiwan; 2 National Institute of Infectious Diseases and Vaccinology, National Health Research Institutes, Miaoli, Taiwan; 3 Graduate Institute of Immunology, National Taiwan University, College of Medicine, Taipei, Taiwan; National Institute of Health, United States of America

## Abstract

Most live-attenuated tetravalent dengue virus vaccines in current clinical trials are produced from Vero cells. In a previous study we demonstrated that an infectious cDNA clone-derived dengue type 4 (DEN-4) virus retains higher genetic stability in MRC-5 cells than in Vero cells. For this study we investigated two DEN-4 viruses: the infectious cDNA clone-derived DEN-4 2A and its derived 3′ NCR 30-nucleotide deletion mutant DEN-4 2AΔ30, a vaccine candidate. Mutations in the C-prM-E, NS2B-NS3, and NS4B-NS5 regions of the DEN genome were sequenced and compared following cell passages in Vero and MRC-5 cells. Our results indicate stronger genetic stability in both viruses following MRC-5 cell passages, leading to significantly lower RNA polymerase error rates when the DEN-4 virus is used for genome replication. Although no significant increases in virus titers were observed following cell passages, DEN-4 2A and DEN-4 2AΔ30 virus titers following Vero cell passages were 17-fold to 25-fold higher than titers following MRC-5 cell passages. Neurovirulence for DEN-4 2A and DEN-4 2AΔ30 viruses increased significantly following passages in Vero cells compared to passages in MRC-5 cells. In addition, more severe DEN-induced hemorrhaging in mice was noted following DEN-4 2A and DEN-4 2AΔ30 passages in Vero cells compared to passages in MRC-5 cells. Target mutagenesis performed on the DEN-4 2A infectious clone indicated that single point mutation of E-Q_438_H, E-V_463_L, NS2B-Q_78_H, and NS2B-A_113_T imperatively increased mouse hemorrhaging severity. The relationship between amino acid mutations acquired during Vero cell passage and enhanced DEN-induced hemorrhages in mice may be important for understanding DHF pathogenesis, as well as for the development of live-attenuated dengue vaccines. Taken together, the genetic stability, virus yield, and DEN-induced hemorrhaging all require further investigation in the context of live-attenuated DEN vaccine development.

## Introduction

The four dengue serotype viruses DEN-1 to DEN-4 (genus *Flavivirus*, family *Flaviviridae)* are single stranded, positive-sense RNA viruses transmitted to humans primarily by *Aedes aegypti* mosquitoes [1]. Their shared RNA genome contains coding sequences for three structural protein genes (core C, precursor membrane prM, and envelope E), seven non-structural protein genes (NS1, NS2A, NS2B, NS3, NS4A, NS4B, NS5), and two flanking non-translating regions (NTRs) [2]. DEN infections in humans result in illnesses ranging from dengue fever (DF) to dengue hemorrhagic fever (DHF) and dengue shock syndrome (DSS). Approximately 50–100 million infections occur annually, including 500,000 cases of DHF and DSS [3], [4], [5], [6], [7]. DEN is endemic in Southeast Asia, where severe forms of DHF and DSS have become major causes of hospitalization among young children [8]. Increases in DEN-related diseases in the past two decades are likely the result of growing human populations, rapid urbanization, the effects of global warming on mosquito vector control, and expanded international travel [9].

There is an urgent need for a safe and effective dengue vaccine. A live-attenuated DEN vaccine would deliver a complete set of protective antigens to achieve long-lasting immunity [7]. The use of live-attenuated tetravalent DEN vaccines against each of the four serotypes would have the potential of minimizing the risk of severe DEN-related diseases [7], [10], [11], [12], [13], [14], [15]. Wild type DEN strains 1 through 4 have been attenuated by serial passages in primary dog and monkey kidney cells [10], [13], [14], and bulk vaccines have been produced using diploid fetal rhesus monkey lung cells (FRhL) or aneuploid African green monkey kidney epithelial cells (Vero) [12], [16], [17]. Results from several clinical trials indicate that each monovalent DEN vaccine is both immunogenic and safe [12], [17]. However, tetravalent vaccine formulation trials have not resulted in predicted responses, with immune imbalance or reactogenicity occurring for certain DEN serotypes [13], [14]. Although an attempt has not been made for production of DEN vaccines, human diploid MRC-5 cells have been used for the production of several live-virus vaccines such as oral polio, rubella, small pox, and varicella zoster [18]. Other vaccine developers have applied cDNA cloning via chimeric virus technology and strategic modifications to generate viruses containing growth restriction phenotypes—for example, DEN-4 with a deletion in 3′ NTR, attenuated 17D yellow fever vaccine, and DEN-2 strain PDK-53 [7], [19], [20], [21], [22], [23], [24], [25], [26], [27], [28], [29], [30]. All of these cDNA-derived candidate vaccines have been produced using Vero cells.

Passages of DEN viruses or their derived chimeras in Vero cells generate mutations that are specific in terms of host cell adaptation, virus attenuation, or other properties [31], [32]. When spot-checking sequences during chimeric DEN-2 PDK-53 vaccine component manufacturing, Stinchcomb et al. (2007) observed the loss of attenuating mutation markers in a number of seed stocks during initial passages in Vero cells; these vaccine seeds were rejected for further use [33]. It is possible that virus passage in certain cells produce host cell-specific mutations that contribute to innate immunity response in vaccinees.

**Figure 1:**
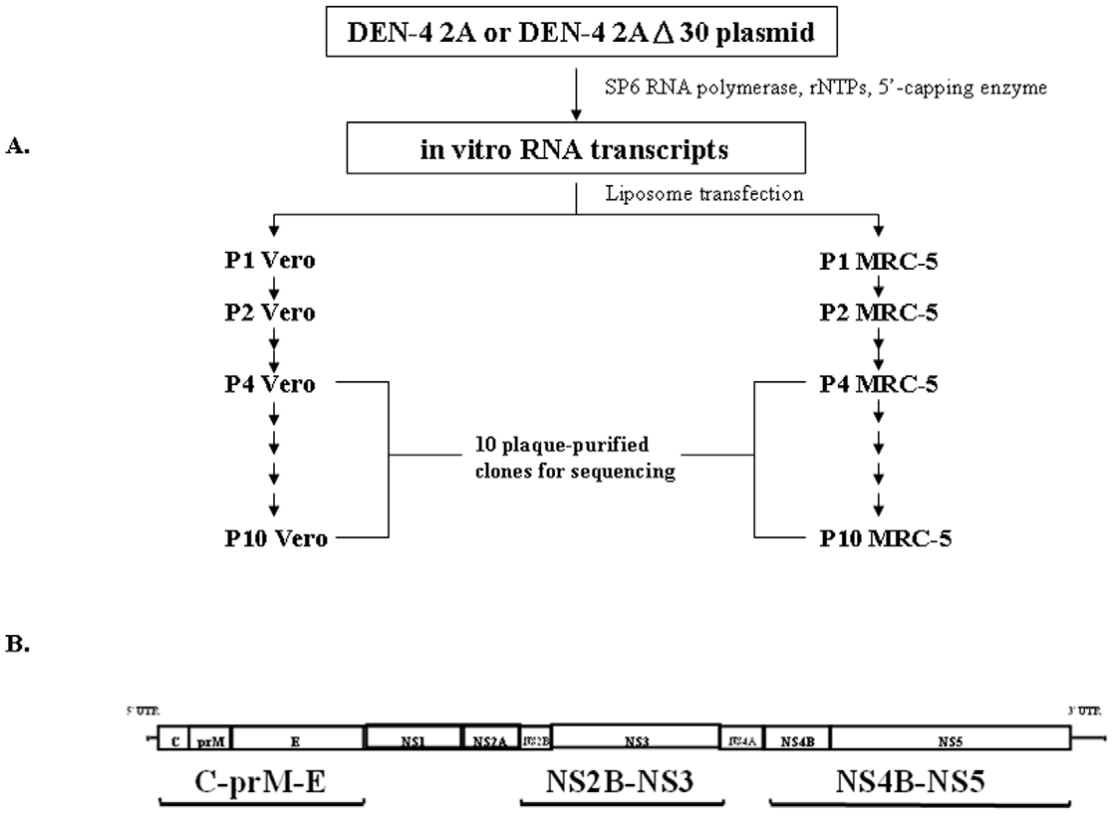
Experiment design. (A) Preparatory passages were performed for 10 cloned DNA-derived DEN-4 2A and 2AΔ30 strain virus plaque-purified clones. Both strains were passaged 10 times each in Vero and MRC-5 cell lines. Virus clones were prepared from discontiguous passages P4 and P10. (B) The dengue virus genome and the sequenced gene fragments C-prM-E, NS2B-NS3 and NS4B-NS5 were used in this study.

We previously demonstrated that the infectious cDNA clone-derived DEN-4 2A virus retains higher genetic stability in MRC-5 cells compared to Vero cells [34]. For the present study we investigated the effects of serial passages in Vero cells and MRC-5 cells on two DEN-4 viruses: a recombinant version of wild type virus DEN-4 2A, and its derived 3′ NCR 30-nucleotide deletion mutant vaccine candidate DEN-4 2AΔ30 [21], [35]. DEN-4 2A and DEN-4 2AΔ30 viruses were generated in Vero and MRC-5 cells via the transfection of in vitro RNA transcripts synthesized using SP6 RNA polymerase (Fig. 1A). For purposes of analyzing genetic mutations that occur during cell passages, we collected ten plaque-purified clones following passages P4 and P10 and sequenced the DEN genomic fragments C-prM-E, NS2B-NS3 and NS4B-NS5 (Fig. 1B), since most mutations described in previous reports occurred in those regions [34].

In addition to focusing on the genetic stability of DEN-4 2A and DEN-4 2AΔ30 viruses following passages in Vero and MRC-5 cells, we also studied associated neurovirulence, neutralizing antibodies, and DEN-induced hemorrhaging in mice. Specifically, DEN neurovirulence attenuation was evaluated in newborn mice and DEN-induced hemorrhaging was examined in an immunocompetent mouse model [36], [37]. Target mutagenesis on DEN4-2A virus E and NS2B proteins were used to confirm the amino acid mutations correlated with mouse hemorrhaging severity. We found additional evidence indicating that (a) the genetic stability of live-attenuated DEN candidate vaccine viruses varies according to the cell line used for vaccine production, and (b) DEN-induced hemorrhaging was much more severe following passages in Vero cells compared to passages in MRC-5 cells.

## Results

### C-prM-E gene mutations resulting from Vero and MRC-5 cell passages

Mutations in the C-prM-E genes of DEN-4 2A viruses resulting from passages in Vero and MRC-5 cells are shown in Table 1. We found 7 nucleotide mutations in the C and E genes following Vero cell passage P4, resulting in 1 amino acid mutation in the C protein (C-F_37_L) and 5 in the E protein (E-R_99_K, E-T_138_P, E-G_427_R, E-V_439_F, E-V_463_L). For viruses obtained following Vero cell passage P10, we found 8 nucleotide mutations in the DEN-4 2A viruses of C-prM-E genes, resulting in 1 amino acid mutation in the C protein (C-F_37_L) and 6 in the E protein (E-R_99_K, E-T_138_P, E-G_328_S, E-G_427_R, E-Q_438_H, and E-V_463_L). Amino acid mutations at E-G_328_S and E-Q_438_H were only detected following P10, with respective mutation frequencies of 50% and 100%. The E gene amino acid mutation at E-V_439_F was only detected following Vero cell passage P4. In contrast, following DEN-4 2A passage P4 in MRC-5 cells we found 3 nucleotide mutations in C-prM-E genes (corresponding to amino acid changes E-E_345_K, E-N_362_K, and E-G_427_R), and 3 mutations following passage P10 (E-E_345_K, E-N_362_K, and E-Q_438_H).

**Table 1:** Nucleotide (nt) and amino acid (aa) changes in C-prM-E fragments from plaque-purified Vero- and MRC-5-passaged DNA-derived DEN-4 2A and DEN-4 2AΔ30 virus clones.

Virus	Cell	Passage Number	Virus Gene Segment	Mutation			
				Frequency	Plaque-purified Clone Number	Nucleotide Position	Amino Acid Position
DEN-4 2A	Vero	4	C	2/10	1, 8	T 210 C	F 37 L
DEN-4 2A	Vero	4	E	4/10	1, 3, 5, 8	G 296 A	R 99 K
DEN-4 2A	Vero	4	E	2/10	4, 9	A 412 C	T 138 P
DEN-4 2A	Vero	4	E	7/10	1, 2, 3, 6, 7, 9, 10	T 1050 A	Silent
DEN-4 2A	Vero	4	E	3/10	4, 8, 10	G 1279 C	G 427 R
DEN-4 2A	Vero	4	E	1/10	9	G 1315 T	V 439 F
DEN-4 2A	Vero	4	E	3/10	2, 5, 7	G 1387 T	V 463 L
DEN-4 2A	Vero	10	C	3/10	1, 3, 8	T 210 C	F 37 L
DEN-4 2A	Vero	10	E	10/10	1, 2, 3, 4, 5, 6, 7, 8, 9, 10	G 296 A	R 99 K
DEN-4 2A	Vero	10	E	8/10	2, 3, 4, 5, 7, 8, 9,10	A 412 C	T 138 P
DEN-4 2A	Vero	10	E	5/10	3, 4, 7, 8, 9	G 982 A	G 328 S
DEN-4 2A	Vero	10	E	8/10	1, 2, 3, 5, 6, 7, 9, 10	T 1050 A	Silent
DEN-4 2A	Vero	10	E	5/10	2, 4, 5, 8, 10	G 1279 C	G 427 R
DEN-4 2A	Vero	10	E	10/10	1, 2, 3, 4, 5, 6, 7, 8, 9, 10	G 1314 T	Q 438 H
DEN-4 2A	Vero	10	E	9/10	1, 2, 3, 5, 6, 7, 8, 9, 10	G 1387 T	V 463 L
DEN-4 2A	MRC-5	4	E	9/10	1, 2, 3, 4, 5, , 7, 8, 9, 10	G 1033 A	E 345 K
DEN-4 2A	MRC-5	4	E	4/10	2, 5, 8, 10	C 1086 A	N 362 K
DEN-4 2A	MRC-5	4	E	2/10	3, 7	G 1279 A	G 427 R
DEN-4 2A	MRC-5	10	E	10/10	1, 2, 3, 4, 5, 6, 7, 8, 9, 10	G 1033 A	E 345 K
DEN-4 2A	MRC-5	10	E	3/10	5, 8, 10	C 1086 A	N 362 K
DEN-4 2A	MRC-5	10	E	1/10	3	G 1314 C	Q 438 H
DEN-4 2AΔ30	Vero	10	E	2/10	1, 8	C 2169 G	R 410 G
DEN-4 2AΔ30	Vero	10	E	8/10	1, 2, 5, 6, 7, 8, 9, 10	T 2231A	Silent
DEN-4 2AΔ30	Vero	10	E	2/10	1, 8	T 2266 C	V 443 A
DEN-4 2AΔ30	Vero	10	E	7/10	1, 2, 6, 7, 8, 9, 10	C 2341 A	T 468 K
DEN-4 2AΔ30	Vero	10	E	4/10	1, 5, 7, 8	A 2370 C	T 491 P

Whereas no C-prM-E gene mutations were identified from DEN-4 2AΔ30 sequencing following Vero cell passage P4 (Table 1), 4 (indicating 5 nucleotide changes) were identified following passage P10 (E-R_410_G, E-V_443_A, E-T_468_K, and E-T_491_P). No C-prM-E mutations for the DEN-4 2AΔ30 virus were found following MRC-5 cell passages P4 or P10 (Table 1). The reduced number of DEN-4 2AΔ30 virus mutations yielded only 4 amino acid changes following Vero cell passage P10; no DEN-4 2AΔ30 virus mutations were found following Vero cell passage P4 or MRC-5 cell passages P4 and P10.

### NS2B-NS3 and NS4B-NS5 gene mutations during Vero and MRC-5 cell passages

DEN-4 2A virus mutations in full-length NS2B-NS3 and NS4B-NS5 genes are shown in Table 2. The P4 Vero cell passage of the DEN-4 2A virus produced only 1 nucleotide change in NS2B-NS3 genes (amino acid mutation NS3-R_418_T); in contrast, 5 nucleotide changes in NS2B-NS3 genes resulted from Vero cell passage P10 of the same virus (mutations NS2B-G_69_R, NS2B-Q_78_H, NS2B-G_108_R, NS2B-A_113_T, and NS3-R_418_T). For the DEN-4 2A virus, no nucleotide mutations were found following MRC-5 cell passages P4 or P10 (Table 2); furthermore, no nucleotide mutations were observed in NS4B-NS5 genes following DEN-4 2A virus passages P4 or P10 in either Vero or MRC-5 cells (Table 2).

**Table 2:** Nucleotide (nt) and amino acid (aa) changes in NS2B-NS3 fragments of plaque-purified Vero- and MRC-5-passaged DNA-derived DEN-4 2A and DEN-4 2AΔ30 virus clones.

Virus	Cell	Passage Number	Virus Gene Segment	Mutation			
				Frequency	Plaque-purified Clone Number	Nucleotide Position	Amino Acid Position
DEN-4 2A	Vero	4	NS3	2/10	3, 8	G 5776 C	R 418 T
DEN-4 2A	Vero	10	NS2B	5/10	2, 4, 5, 6, 9	G 4338 C	G 69 R
DEN-4 2A	Vero	10	NS2B	10/10	1, 2, 3, 4, 5, 6, 7, 8, 9, 10	G 4367 T	Q 78 H
DEN-4 2A	Vero	10	NS2B	7/10	1, 3, 4, 7, 8, 9, 10	G 4455 C	G 108 R
DEN-4 2A	Vero	10	NS2B	3/10	2, 5, 6	G 4470 A	A 113 T
DEN-4 2A	Vero	10	NS3	5/10	3, 4, 5, 8, 10	G 5776 C	R 418 T
DEN-4 2AΔ30	Vero	10	NS2B	10/10	1, 2, 3, 4, 5, 6, 7, 8, 9, 10	C 4224 A	P 31 T
DEN-4 2AΔ30	Vero	10	NS2B	10/10	1, 2, 3, 4, 5, 6, 7, 8, 9, 10	G 4295 C	E 54 D
DEN-4 2AΔ30	Vero	10	NS2B	3/10	1, 5, 8	A 4344 C	S 71 R
DEN-4 2AΔ30	Vero	10	NS2B	10/10	1, 2, 3, 4, 5, 6, 7, 8, 9, 10	G 4356 C	E 75 Q
DEN-4 2AΔ30	Vero	10	NS2B	6/10	1, 2, 3, 5, 8, 9	G 4359 A	V 76 M

For DEN-4 2AΔ30, Vero cell passage P4 did not produce any sequence mutations in NS2B-NS3 genes, but mutations at NS2B-P_31_T, NS2B-E_54_D, NS2B-S_71_R, NS2B-E_75_Q, and NS2B-V_76_ M were noted following P10 (mutation frequencies 100%, 100%, 30%, 100%, and 60%, respectively) (Table 2). No nucleotide or amino acid mutations were observed in the NS3 gene of the DEN-4 2AΔ30 virus following Vero cell passage P10, nor in NS2B-NS3 genes following MRC-5 cell passage P4 or P10 (Table 2). Finally, results from sequencing the NS4B-NS5 region indicate no nucleotide mutations for either DEN-4 2A or DEN-4 2AΔ30 following Vero or MRC-5 cell passages.

### DEN-4 2A and DEN-4 2AΔ30 virus titers following Vero and MRC-5 cell passages

**Figure 2:**
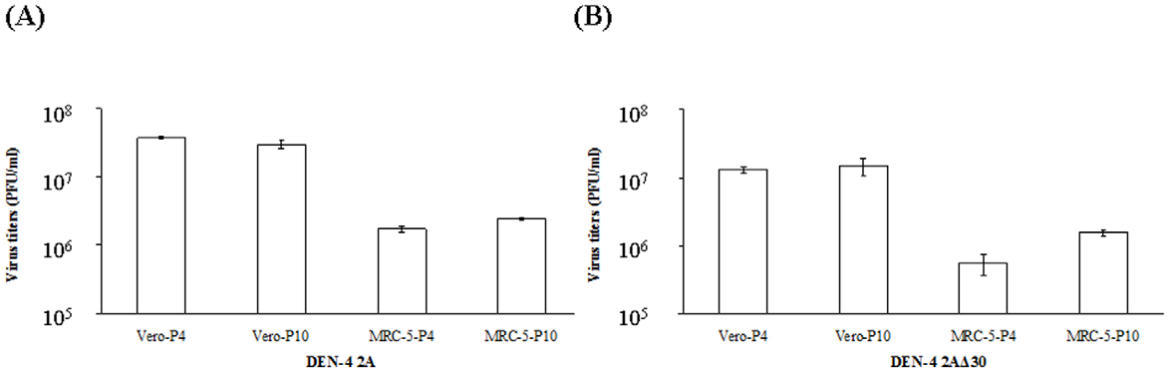
Virus growth properties in Vero and MRC-5 cells. Shown are the highest virus titers following passages P4 and P10 in Vero and MRC-5 cells for (A) DEN-4 2A (B) DEN-4 2AΔ30.

We also investigated the effects of Vero and MRC-5 cell passages on DEN-4 2A and DEN-2AΔ30 virus growth. Virus titer data (measured at 8 dpi) for DEN-4 2A and DEN-4 2A Δ30 following P4 and P10 passages in both cell types are shown in Figures 2A and 2B. The data indicate cell growth following DEN virus infection at a MOI of 0.01, with no significant differences between the two viruses following Vero cell passages P4 and P10. Also for both viruses, a slight increase in virus titers was found for MRC-5 cell passage P10 compared to P4. Titers of both viruses following Vero cell passages were between 17-fold to 25-fold higher compared to titers measured after MRC-5 cell passages.

### DEN-4 2A and DEN-4 2AΔ30 virus neurovirulence following Vero and MRC-5 cells passages

**Figure 3:**
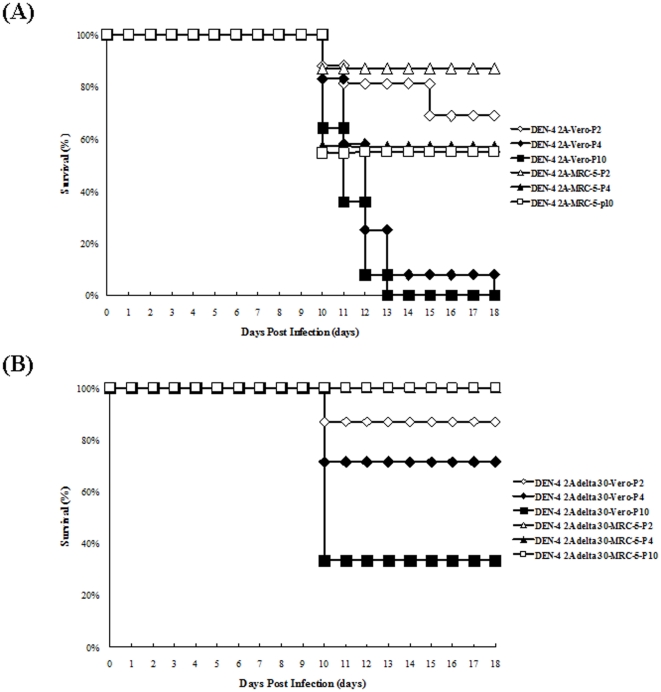
Neurovirulence assay results for newborn mice injected with cloned DNA-derived DEN-4 2A and DEN-4 2AΔ30 virus strains following passages in Vero and MRC-5 cells. Shown are survival rates for newborn mice infected with (A) DEN-4 2A and (B) DEN-4 2AΔ30.

To further evaluate DEN-4 2A and DEN-4 2AΔ30 neurovirulence following Vero and MRC-5 cell passages, we intracranially inoculated a group of newborn ICR mice with 10^4^ PFU/30 µl of either virus stock. Survival data are shown in Figures 3A and 3B. As shown, higher neurovirulence was observed in both the DEN-4 2A and the DEN-4 2AΔ30 viruses following passages P2, P4 and P10 in Vero cells. In contrast, a significant less extent of mouse neurovirulence was observed in the DEN-4 2A virus following passage P2, P4, and P10 in MRC-5 cells. The DEN-4 2AΔ30 viruses passaged in MRC-5 cells did not show any neurovirulence. Overall, neurovirulence for DEN-4 2A and DEN-4 2AΔ30 viruses increased significantly following passages in Vero cells compared to passages in MRC-5 cells.

### DEN-induced mouse hemorrhage pathogenicity following DEN-4 2A and DEN-4 2AΔ30 passages in Vero and MRC-5 cells

**Figure 4:**
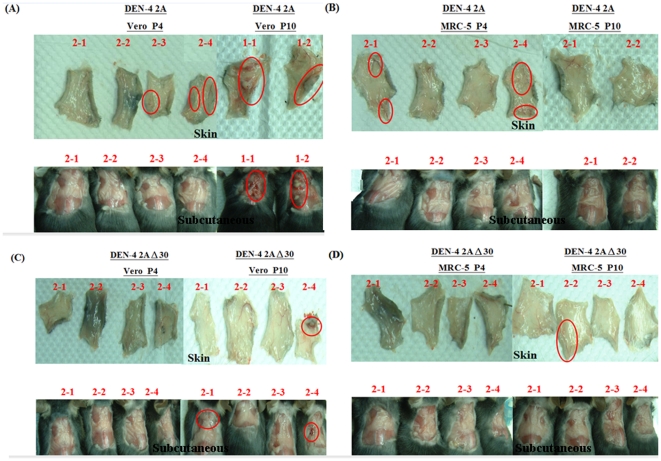
Hemorrhage assessment of immunocompetent C57BL/6 mice. DEN-4 2A viruses following passages in (A) Vero and (B) MRC-5 cells and DEN-4 2AΔ30 viruses following passages in (C) Vero and (D) MRC-5 cells were intradermally injected with 4×10^7^ PFU DENV (in 0.4 ml) at four sites on the upper back. To determine degree of hemorrhaging, mice were sacrificed at 3 days post-inoculation, and subcutaneous tissues in the back, abdomen, axillary areas, and thorax were exposed.

We examined DEN-induced hemorrhaging in an immunocompetent mouse model [36], [37]. C57BL/6 mice were injected intradermally with DEN-4 2A or DEN-4 2AΔ30 viruses that had been passaged in Vero or MRC-5 cells (P4 and P10). Data from two independent experiments are shown in Table 3. DEN-induced hemorrhaging was observed in epidermal and subcutaneous tissues at day 3 (Fig. 4). Between Vero cell passages P4 and P10, average hemorrhage rate (± S.D.) increased from 58±11% to 100% for the DEN-4 2A virus, and from 0% to 45±7% for the DEN-4 2AΔ30 virus. In contrast, between MRC-5 cell passages P4 and P10, the average rate changed from 58±11% to 33±46% for DEN-4 2A, and 17±23% to 23±3% for DEN-4 2AΔ30. In other words, more severe DEN-induced hemorrhaging occurred following DEN-4 2A and DEN-4 2AΔ30 passages in Vero cells compared to the same passages in MRC-5 cells.

**Table 3:** Hemorrhage assessment in immunocompetent C57BL/6 mice of passage 4 and 10, DEN-4 2A and DEN-4 2AΔ30 in Vero cells and MRC-5 cells.

Virus Strain	Cell Line	Passage Number	Mouse No.	DEN-induced hemorrhaging in mice			
				Skin	Subcutaneous	% Hemorrhage Development	Average % of Mice withHemorrhage Development
DEN-4 2A	Vero	4	1-1	−	−	66%	58±11%
		4	1-2	++	+		
		4	1-3	+++	++		
		4	2-1	−	−	50%	
		4	2-2	−	−		
		4	2-3	+	−		
		4	2-4	+	−		
		10	1-1	++	+	100%	100±0%
		10	1-2	++	+		
		10	2-1	−	++	100%	
		10	2-2	++	+		
		10	2-3	+	−		
		10	2-4	−	+		
		10	2-5	++	+		
	MRC-5	4	1-1	−	−	66%	58±11%
		4	1-2	++	+		
		4	1-3	++	+		
		4	2-1	+	−	50%	
		4	2-2	−	−		
		4	2-3	−	−		
		4	2-4	+	−		

Virus Strain	Cell Line	Passage Number	Mouse No.	DEN-induced hemorrhaging in mice			
				Skin	Subcutaneous	% Hemorrhage Development	Average % of Mice withHemorrhage Development
DEN-4 2A	MRC-5	10	1-1	−	++	66%	33±46%
		10	1-2	−	++		
		10	1-3	−	−		
		10	2-1	−	−	0%	
		10	2-2	−	−		
DEN-42AΔ30	Vero	4	1-1	−	−	0%	0±0%
		4	1-2	−	−		
		4	1-3	−	−		
		4	2-1	−	−	0%	
		4	2-2	−	−		
		4	2-3	−	−		
		4	2-4	−	−		
		10	1-1	+	−	40%	45±7%
		10	1-2	−	−		
		10	1-3	−	−		
		10	1-4	+	+		
		10	1-5	−	−		
		10	2-1	−	+	50%	
		10	2-2	−	−		
		10	2-3	−	−		

Virus Strain	Cell Line	Passage Number	Mouse No.	DEN-induced hemorrhaging in mice			
				Skin	Subcutaneous	% Hemorrhage Development	Average % of Mice withHemorrhage Development
DEN-42AΔ30	MRC-5	4	1-1	−	−	33%	17±23%
		4	1-2	+	−		
		4	1-3	−	−		
		4	2-1	−	−	0%	
		4	2-2	−	−		
		4	2-3	−	−		
		4	2-4	−	−		
		10	1-1	−	−	20%	23±3%
		10	1-2	+	−		
		10	1-3	−	−		
		10	1-4	−	−		
		10	1-5	−	−		
		10	2-1	−	−	25%	
		10	2-2	+	−		
		10	2-3	−	−		
		10	2-4	−	−		

Rates represent hemorrhage percentage in each independent experiment. A three-level bleeding scoring low (“+”), medium (“++”), high (“+++”), and no bleeding (“−“) was used to indicate the severity of hemorrhaging between different experimental cases.

### DEN-induced mouse hemorrhage pathogenicity by DEN-4 2A mutant of E-T_138_P, E-G_328_S, E-Q_438_H, E-V_463_L, NS2B-G_69_R, NS2B-Q_78_H, NS2B-G_108_R, or NS2B-A_113_T

Target mutagenesis by using DEN-4 2A infectious clone was finally conducted to examine the mutations of E-T_138_P, E-G_328_S, E-Q_438_H, E-V_463_L, NS2B-G_69_R, NS2B-Q_78_H, NS2B-G_108_R, and NS2B-A_113_T on DEN-induced mouse hemorrhage pathogenicity. Data are shown in Table 4 and Table 5 for E and NS2B mutants, respectively. DEN-induced hemorrhaging by DEN-4 2A mutant viruses was observed in epidermal and subcutaneous tissues at day 3 compared to wild type DEN-4 2A virus. Recombinant DEN-4 2A with single mutations at E-Q_438_H, E-V_463_L, NS2B-Q_78_H, and NS2B-A_113_T had increased severity of DEN-induced mouse hemorrhaging as compared to the wild-type DEN-4 2A viruses. Collectively, our data suggest that the E-Q_438_H, E V_463_L, NS2B-Q_78_H and NS2B-A_113_T mutations, which were acquired from DEN-4 2A virus passaged in Vero cells, enhanced hemorrhaging in the immunocompetent mouse model, irrespective of other E and NS2B mutations acquired during DEN-4 2A virus passaged in Vero cells.

**Table 4:** Hemorrhage assessment in immunocompetent C57BL/6 mice of DEN-4 2A E gene single amino acid mutant viruses.

Virus Strain	SubstitutionMutationViruses	Mouse No.	Severity of Hemorrhage		% of Hemorrhage Development
			Skin	Subcutaneous	
DEN-4 2A	Wild type	1-1	+	−	20%
		1-2	−	−	
		1-3	−	−	
		1-4	−	−	
		1-5	−	−	
	E-T_138_P	2-1	++	−	40%
		2-2	+	−	
		2-3	−	−	
		2-4	−	−	
		2-5	−	−	
	E-G_328_S	3-1	−	−	20%
		3-2	−	−	
		3-3	−	−	
		3-4	−	−	
		3-5	+	−	
	E-Q_438_H	4-1	−	−	60%
		4-2	−	+	
		4-3	−	−	
		4-4	+	+	
		4-5	++	−	
	E-V_463_L	5-1	−	−	80%
		5-2	+++	−	
		5-3	+	+	
		5-4	+	−	
		5-5	++	−	

Rates represent hemorrhage percentage in each independent experiment. A three-level bleeding scoring low (“+”), medium (“++”), high (“+++”), and no bleeding (“−“) was used to indicate the severity of hemorrhaging between different experimental cases.

**Table 5:** Hemorrhage assessment in immunocompetent C57BL/6 mice of DEN-4 2A NS2B gene single amino acid mutant viruses.

Virus Strain	SubstitutionMutationViruses	Mouse No.	Severity of Hemorrhage		% of Hemorrhage Development
			Skin	Subcutaneous	
DEN-4 2A	Wild type	1-1	−	−	40%
		1-2	+	−	
		1-3	+	−	
		1-4	−	−	
		1-5	−	−	
	NS2B-G_69_R	2-1	−	−	20%
		2-2	++	−	
		2-3	−	−	
		2-4	−	−	
		2-5	−	−	
	NS2B-Q_78_H	3-1	−	−	60%
		3-2	++	+	
		3-3	+	+	
		3-4	−	++	
		3-5	−	−	
	NS2B-G_108_R	4-1	−	−	0%
		4-2	−	−	
		4-3	−	−	
		4-4	−	−	
		4-5	−	−	
	NS2B-A_113_T	5-1	+	−	100%
		5-2	++	++	
		5-3	++	++	
		5-4	+	+	
		5-5	+	+	

Rates represent hemorrhage percentage in each independent experiment. A three-level bleeding scoring low (“+”), medium (“++”), high (“+++”), and no bleeding (“−”) was used to indicate the severity of hemorrhaging between different experimental cases.

## Discussion

Since live-attenuated vaccines require limited seed virus passage levels to prevent unsafe reversion, seed viruses must maintain their genetic stability during cell passages as part of the vaccine manufacturing process. Based on 74% of DEN genome sequencing (including all C-prM-E structure protein genes and full-length NS2B, NS3, NS4B, and NS5 genes), our results indicate stronger genetic stability for the infectious cDNA clone-derived viruses DEN-4 2A or DEN-4 2AΔ30 following passages in MRC-5 cells compared to passages in Vero cells. This corresponds to significantly lower DENpol error rates when the DEN virus is used for genome replication.

Pugachev et al.'s (2004) method for analyzing plaque-purified clones following different cell passages provides a systematic means for qualitatively and quantitatively examining the genetic stability of vaccine viruses [38]. In an earlier study we examined DEN-4 2A virus mutations following Vero and MRC-5 cell passages by sequencing multiple clones of DNA fragments synthesized from DEN-4 2A RNA by RT-PCR [34]. This approach may not accurately identify viable viruses with identical mutations by sequencing multiple virus stock clones, but we found that plaque purification supports the selection of viable mutant viruses for sequencing multiple virus clones. We found that the number of mutations detected by plaque-purified clone virus sequencing was generally larger than the number detected by sequencing multiple virus clones. In our previous study, the sequencing of multiple DEN-4 2A virus clones supported the identification of mutations occurring at E-G_104_C (70%), E-F_108_I (60%), E-G_427_R (20%), E-V_439_F (10%), and E-V_463_L (10%) following passage P3 in Vero cells [34]. In the present study, the sequencing of 10 plaque-purified DEN-4 2A virus clones during passage P4 (equivalent to P3 in our earlier work) in Vero cells resulted in the detection of mutations at C-F_37_L (20%), E-R_99_K (40%), E-T_138_P (20%), E-G_427_R (30%), E-V_439_F (10%), and E-V_463_L (30%). The differences may be due to the low sensitivity associated with sequencing multiple clones compared to mutations detected by sequencing plaque-purified clones.

Differences in nucleic acid mutations detected after DEN-4 2A and DEN-4 2AΔ30 passages P4 and P10 in Vero and MRC-5 cells are shown in Tables 1 and 2. In our genetic stability analyses we did not include mutations induced by SP6 RNA polymerase when synthesizing RNA transcripts in vitro, or early stage mutations from passages P1, P2, and P3. In a separate study we used direct sequencing to analyze the full-length DEN genomes of (a) the DEN-4 2A virus following passage P1 in Vero cells and (b) the DEN-2AΔ30 virus following passage P2 in Vero cells; we observed 6 nucleotide changes in the former and 16 in the latter (data not shown). If these mutations were mistakes resulting from the introduction of SP6 RNA polymerase during a single round of DNA-dependent RNA synthesis, then our error rates would have been 5.63×10^−4^ per nucleotide copied for the DEN-4 2A infectious clone (6 misincorporations per genomic RNA molecule with a length of 10,649 nucleotides), and 1.5×10^−3^ per copied nucleotide for the DEN-4 2AΔ30 infectious clone (16 misincorporations per genomic RNA molecule with a length of 10,619 nucleotides). These results are comparable to the SP6 RNA polymerase error rate of 1.34×10^−4^ per nucleotide reported by Pugachev et al. [38]. To calculate estimated error rates for DEN virus RNA polymerase in C-prM-E, NS2B-NS3 and NS4B-NS5 genes, we divided the number of DENpol mistakes by the number of sequenced full genome equivalents, the estimated number of RNA synthesis rounds during one plaque formation cycle, and the number of plaque purification steps for each clone [38]. As shown in Table S1, we identified 37 and 28 DENpol mistakes in the DEN-4 2A virus during Vero cell passages P4 and P10 for the C-prM-E and NS2B-NS3 genes, respectively; in contrast, only 2 C-prM-E and 0 NS2B-NS3 mistakes were found following MRC-5 cell passages P4 and P10. The estimated C-prM-E gene error rates following DEN-4 2A Vero and MRC-5 cell passages were 1.05–1.16×10^−6^ and 6.29–7.30×10^−8^ per copied nucleotide per genomic RNA molecule (10,649 nt). Compared to MRC-5 cell passages, the estimated C-prM-E gene error rate in the DEN-4 2A virus increased 17.5-fold following Vero cell passages. Estimated C-prM-E gene error rates for the DEN-4 2AΔ30 virus following Vero and MRC-5 passages were 6.57–7.26×10^−7^ and 0 per copied nucleotide, respectively. Regarding NS2B-NS3 gene mutations during Vero cell passages, estimated error rates were 7.98–8.81×10^−7^ and 1.11–1.23×10^−6^ per copied nucleotide for DEN-4 2A and DEN-4 2AΔ30 viruses, respectively. In all cases, DENpol error rates for both viruses were significantly higher following Vero cell passages compared to MRC-5 cell passages. However, we can not rule out other cellular factors interacted with viral factors and play a role in the selection of specific mutations.

The replication kinetics of DEN-4 2A and DEN-4 2AΔ30 viruses have been investigated in Vero and MRC-5 cells which are now being used to develop several human vaccines [18]. The viral yields of DEN-4 2A and DEN-4 2AΔ30 viruses produced in MRC-5 cells on microcarriers (at P4 or P10) were approximately 10-fold lower compared to those in Vero cells with more amino acid mutations during the , respectively (Table 2). One research team that has introduced mutations into the 3′-NTR of DEN (using the DEN-4 2AΔ30 infectious cDNA clone for purposes of attenuating vaccine candidates) describes the DEN-4 2AΔ30 cDNA clone as balancing between attenuation and immunogenicity in a non-human primate model [11], [15], [21], [35], [39]. We used newborn ICR mice to evaluate the attenuation of neurovirulence of DEN-4 2A and DEN-4 2AΔ30 viruses following passages P2, P4, and P10 in Vero or MRC-5 cells. In a previous study, Huang et al. evaluated the neurovirulence of DEN-4 1036 virus (collected from an Indonesian child with dengue fever) by injecting 10^4^ PFU of the virus intracranially into newborn ICR mice; average survival time was 8.6±0.6 days [26]. Average survival times for newborn ICR mice intracranially injected with passaged DEN-4 2A and DEN-4 2AΔ30 viruses were longer than that for DEN-4 1036-treated mice, indicating less neurovirulence for the DEN-4 2A and DEN-4 2AΔ30 viruses. Neurovirulence for DEN-4 2A and DEN-4 2AΔ30 viruses increased significantly following passages in Vero cells compared to passages in MRC-5 cells.

In addition to being the major determinant of tropism and virulence, the DEN virus E protein is the primary target of neutralizing antibodies. The P10 Vero cell passages of the two DEN-4 infectious clone viruses resulted in mutations at residues E-R_99_K, E-T_138_P, E-G_328_S, E-G_427_R, E-Q_438_H, and E-V_463_L in the DEN-4 2A virus, and residues E-R_410_G, E-V_443_A, E-T_468_K, and E-T_491_P in the DEN-4 2AΔ30 virus. In contrast, the P10 MRC-5 cell passages produced mutations at residues E-E_345_K, E-N_362_K, and E-Q_438_H of the DEN-4 2A virus only—no DEN-4 2AΔ30 virus mutations were observed. Our results using target mutagenesis at E-Q_438_H and E-V_463_L to generate mutant viruses had increased severity of DEN-induced mouse hemorrhaging (Table 4). The E-Q_438_H amino acid mutation, which is located in the second helix domain (E-H2) of the E protein stem region, was detected following passage P10 only (100% mutation frequency). The E-V_463_L mutation in the DEN-4 2A virus resulting from the P10 Vero cell passage is located in the N-terminus of the first (E-T1) helix of the E protein transmembrane domain (TMD), which also contains an endoplasmic reticulum retention signal. Our results indicate more severe DEN-induced hemorrhages in mice following DEN-4 2A and DEN-4 2AΔ30 passages in Vero cells, but not following passages in MRC-5 cells.

A cluster of NS2B mutations also appeared during Vero cell passage P10 in both DEN-4 2A and DEN-4 2AΔ30 viruses (Tables 3 and 4). Flavivirus-specified protease activity for cytosol cleavages at dibasic sites in polyproteins requires both NS2B and NS3 [40], [41]. The dual-component NS2B-NS3 protease executes most of this segmentation at the NS2A/NS2B, NS2B/NS3, NS3/NS4A, and NS4B/NS5 junctions. The cleavage mediated by NS2B and NS3 is an essential step in viral replication. The NS3 N-terminus encodes the enzymatic core, while a hydrophilic core within NS2B (NS2Bc) provides an essential cofactor function [42], [43]. NS2Bc unravels to increase the basal proteolytic activity of NS3 protease by 3,300-fold to 7,600-fold [44]. It was reported that the C-terminal region of NS2Bc (residues 67 to 95) plays a substrate-binding role in the proteolytic activity of NS3 protease [45]. The NS2B-G_69_R and NS2B-Q_78_H mutations from DEN-4 2A passages in Vero cells and the NS2B-S_71_R, NS2B-E_75_Q, and NS2B-V_76_M mutations from DEN-2A Δ30 passages in Vero cells are located in the C-terminal region of NS2Bc. Target mutagenesis on NS2B mutant viruses also indicated that single point mutation of NS2B-Q_78_H and NS2B-A_113_T imperatively increased mouse hemorrhaging severity. As the NS2B is a cofactor for NS3 protease activation, the mutation of NS2B-Q_78_H is located in a hydrophobic stretch of NS2B residues Gly^70^-Glu^81^, which located in a hydrophilic cofactor domain as previously reported for all flaviviruses [46]. The side chain group changed from amide group to imidazole group, and the electricity change from no charge to positive charge of NS2B-Q_78_H may change the NS2B cofactor efficiency to affect the NS3 protease activity [47]. The NS2B-Q_78_H and NS2B-A_113_T had the same virus replication patterns in human microvascular endothelial (HMEC-1) cells with the wild type virus, and these mutations had no influence on affecting the cell survival signaling or introduce cell death pathway in the infected cells which then leads to hemorrhaging phenotypes (data not shown). The relationship of the NS2B/NS3 protease interactions contributed to DEN-induced hemorrhaging in mice is still unknown. The virus-host cell mechanism underlying DHF is not fully understood, and the relationship between amino acid mutations acquired during Vero cell passage and enhanced DEN-induced hemorrhages in mice may be important for understanding DHF pathogenesis, as well as for the development of live–attenuated dengue vaccines.

## Materials and Methods

### Cells and Media

Vero, Vero E6, and MRC-5 cells were obtained from the Bioresource Collection and Research Center (BCRC) of the Food Industrial Research and Development Institute, Hsinchu, Taiwan. Vero cells (African green monkey kidney cells) were derived from ATCC CCL-81 (BCRC number: 60013). Vero E6 cells (BCRC number: 60476; ATCC CRL-1586), derived from VERO 76 cells (ATCC CRL-1587), were cloned by the microtiter plate dilution method. MRC-5 cells (human embryonal lung fibroblasts) were derived from ATCC CCL-171 (BCRC number: 60023). Vero, Vero E6, and MRC-5 cells were grown in Dulbecco's Modified Essential Medium (DMEM) (Invitrogen) supplemented with 10% heat-inactivated fetal bovine serum (FBS) and 100 U/ml of penicillin G sodium-streptomycin (Invitrogen). Vero and MRC-5 cells were also cultured in M-VSFM serum free medium (Biogrow) with 100 U/ml of penicillin G sodium-streptomycin. No prior adaptation in reduced serum concentrations was required for the serum free cultures. Trypsin inhibitor (GIBCO) was used at 0.25% for cell detachment to protect cell damage by trypsin treatment under serum-free conditions.

### Viruses

Stock viruses were prepared from the supernatants of infected C6/36 cells grown in Hank's MEM medium (GIBCO) plus supplements. Plasmids of DEN-4 2A and its 3′NCR deletion mutant DEN-4 2AΔ30 contained full-length genomic sequences. Plasmids were linearized by cleavage with the *Kpn* I restriction enzyme and added to a transcription reaction mixture (Promega) containing m^7^G(5′)ppp(5′)G (Merck) for a cap addition at the RNA 5′ end. After incubation at 37°C for 1.5 h, the RNA product was purified with TRIzol reagent (Invitrogen) according to the manufacturer's instructions. Prior to RNA transfection, subconfluent Vero cells and MRC-5 cells in 6-well plates were rinsed once with serum-free medium and covered with 0.3 ml of Opti-MEM medium (Invitrogen) per well. The transfection mixture was prepared by adding 6 µl of DMRIE-C reagent (Invitrogen) to 1 ml of Opti-MEM medium prior to mixing with 10 µg of the RNA product. This mixture was added directly to the cell monolayer. After 18 h of incubation at 37°C, either DMEM+10% FBS or M-VSFM medium was added to each well. Culture supernatants were collected 8 days post-transfection. All virus stocks were stored at −80°C until further analysis.

Virus titers were determined by plaque assays of a Vero E6 cell line. To prepare high-DENV titers (up to 10^9^ plaque-forming units/ml [PFU/ml]), virus supernatant was concentrated by centrifugation with a Centriplus device (10-kDa cutoff) (Amicon; Millipore). To confirm a homogenous virus population, biological clones of passage regimens were generated via single rounds of plaque purification in Vero or MRC-5 cells. Medium 199 (Gibco) containing 3% FBS was used for plaque purification in six-well culture plates (agarose overlays with neutral red staining). Agarose plugs were carefully removed to avoid disturbing the monolayers. Each selected clone was propagated once in Vero or MRC-5 cells to confirm viability.

### Microcarrier cultures

Cytodex 1 microcarriers (Amersham Biosciences) were prepared according to the manufacturer's instructions. Briefly, microcarriers were immersed in PBS for at least 3 h and autoclaved for 15 min before each experiment. Autoclaved microcarriers were washed twice with culture medium. Bellco spinner flasks were used in the experiments at a working volume of 50 ml. Flasks were stirred at 60 rpm and incubated with 5% CO_2_ at 37°C. Cells were detached from tissue culture flasks using trypsin-EDTA, and transferred to flasks containing 2 g/l Cytodex 1 microcarriers. Initial cell densities were 3×10^5^ cells/ml in serum-containing cultures, and 6×10^5^ cells/ml in serum-free cultures. Cell cultures were infected 3 days post-inoculation; 70% of the medium was replaced with fresh medium containing virus inoculum. Inoculation continued without further medium replacement or supplement addition. Virus titers were determined after each passage, with the subsequent culture infected at 0.01 MOI. Microcarrier cultures were used for the P4 to P10 passages from which we gathered mutation data.

### Cell density and virus titer determination

Numbers of cells attached to microcarriers were determined by nuclei staining. Briefly, a 1 ml sample of microcarrier culture was centrifuged at 200 *g* for 5 min to remove supernatant. Pellets were treated with 1 ml 0.1 M citric acid containing 0.1% (w/v) crystal violet and incubated at 37°C for 1 h. Released nuclei were counted in a hemacytometer. Virus titer was determined by 10-fold serial dilutions of culture supernatant in duplicate Vero-E6 cell monolayer infections in 6-well plates. After incubation for 1 h at 37°C, 4 ml of medium containing 1× EMEM (Invitrogen), 1.1% methylcellulose, and 100 U/ml of penicillin G sodium-streptomycin were added to each well. Virus plaques were stained with 1% crystal violet dye 6 days following incubation. Infectivity titers were determined in PFU/ml.

### Plaque purification

Monolayer Vero cells individually infected with tenfold serial dilutions of infectious clone cDNA-derived DEN-4 2A-Vero-P4, DEN-4 2A-Vero-P10, DEN-4 2A-MRC-5-P4, DEN-4 2A-MRC-5-P10, DEN-4 2AΔ30-Vero-P4, DEN-4 2AΔ30-Vero-P10, DEN-4 2AΔ30-MRC-5-P4, and DEN-4 2AΔ30-MRC-5-P10 harvested from passaged cell lines were cultured in 4 ml of Eagle MEM containing 2.5% low-melting agar. Ten variant plaques derived from each sample were collected from monolayer Vero cells 7 days post-infection. At 5 days post-infection, cells were dyed with Eagle MEM (2 ml) containing 2.5% low-melting agar and 1× neutral red solution. These samples were used to infect Vero and MRC-5 cells. Amplified plaque-purified clones were used to sequence and analyze mutation sites.

### Plaque-purified clone sequencing

Three DNA fragments were synthesized from DEN-4 RNA by RT-PCR using Platinum® *Pfx* DNA polymerase and three forward and reverse primer pairs: (i) W01F/W02R, (ii) W16F/W30R, and (iii) W20F/W08R. The DNA products were purified using a Gel/PCR DNA fragment extraction kit (Geneaid, Taiwan). The nucleotide sequences of each fragment were determined by Mission Biotech Inc., Taipei, Taiwan. Lasergene software (v. 6.0) was used to align sequences in the W01F/W02R (2,612-bp), W16F/W30R (3,009-bp), and W20F/W08R regions (4,283-bp) to generate a consensus sequence for each fragment.

### DEN genomic mutation sequencing following Vero and MRC-5 cell passages

DEN-4 infectious clone-derived viruses were generated by the transfection of in vitro RNA transcripts synthesized using SP6 RNA polymerase with two infectious full-length cDNA clones of DEN-4 2A and DEN-4 2AΔ30 passaged in Vero and MRC-5 cells. Ten consecutive passages (P1–P10) were investigated to determine the genetic stability of viruses propagated by the two cell types. To analyze genetic mutations that occurred during cell passages, ten plaque-purified clones collected from virus stocks after passages P4 and P10 were subjected to the genetic sequencing of three DEN genomic fragments (C-prM-E, NS2B-NS3 and NS4B-NS5), based on the rationale that in a previous study most mutations occurred in these regions [34]. As described by Pugachev et al. [38] for four chimeric yellow fever-DEN virus vaccine candidates, it is possible to determine the RNA polymerase fidelity of two infectious DEN-4 viruses propagated in Vero and MRC-5 cells.

### Estimated dengue virus RNA polymerase error rates

Estimated error rates for DEN virus RNA polymerase in C-prM-E, NS2B-NS3, and NS4B-NS5 genes were calculated by dividing the number of DENpol mistakes by the number of sequenced full genome equivalents, the estimated number of rounds of RNA synthesis during one plaque formation, and the number of plaque purification steps for each clone, as previously described [38]. The numbers of sequenced full genome equivalents to the three genomic fragments were 2.2 kb for C-prM-E, 2.2 kb for NS2B-NS3, and 3.4 kb for NS4B-NS5. As DEN4-2A and DEN4-2AΔ30 virus growth reached peak titers of ∼10^7^ PFU/ml in Vero cells and ∼10^6^ PFU/ml in MRC-5 cells, a plaque pick of approximately 500 µl resulted in ∼5×10^6^ infectious particles being produced in Vero cells and ∼5×10^5^ infectious particles being produced in MRC-5 cells. Assuming that 100 to 1,000 times additional RNA molecules are synthesized during flavivirus replication [38], the estimated numbers of RNA synthesis rounds for a single plaque formation are 29–32 in Vero cells and 25–29 in MRC-5 cells.

### Target mutagenesis, construction of DEN-4 2A E-T_138_P, E-G_328_S, E-Q_438_H, E-V_463_L, NS2B-G_69_R, NS2B-Q_78_H, NS2B-G_108_R, and NS2B-A_113_T infectious cDNA clones, and recovery of mutant viruses

The infectious clones DEN-4 2A, which contain infectious DENV cDNA corresponding to the anti-genome of the DENV-4 vaccine candidate strain 814669, have been described elsewhere [21]. The clone-derived virus, DEN-4 2A, exhibits the same phenotypes as the DENV-4 vaccine candidate strain 814669 virus and was used as wt control. Target mutagenesis generating the mutant cDNA clones were performed by using overlapped PCR method. To construct DEN-4 2A E-T_138_P infectious cDNA clones, PCR fragments containing corresponding mutations were amplified by two rounds of PCR reactions. The first round was done by using primer pairs *Hpa*I-f : 5′-TGATTGGATTCAGGAAGGAG-3′ and T_138_P-r: 5′-ACAACCACTGGGTATTCAAG-3′, and T_138_P-f: 5′-CTTGAATACCCAGTGGTTGT-3′(this changed E gene amino acid no. 138 from Thr to Pro; from ACA to CCA ) and *Nsi*I-r 5′-AGTCCACTTCTGTGGCTCCA-3′ for construction of DEN-4 2A E-T_138_P. The second round was done by using the same primer pairs *Hpa*I-f : 5′-TGATTGGATTCAGGAAGGAG-3′ and *Nsi*I-r 5′-AGTCCACTTCTGTGGCTCCA-3′. The 1,412 bp *Hpa*I-*Nsi*I PCR fragments were cloned into the pJET1.2/blunt cloning vector (Fermentas Life Sciences Corp.) for amplification. A 1,412 bp region flanked by *Hpa*I and *Nsi*I restriction enzyme sites in DEN-4 2A infectious clone was replaced with *Hpa*I-*Nsi*I fragment derived from confirmed clones, which contained E-T_138_P mutation in DEN-4 2A infectious clones. To construct DEN-4 2A E-G_328_S, E-Q_438_H, E-V_463_L, infectious cDNA clones, PCR fragments containing corresponding mutations were amplified by two rounds of PCR reactions. The first round was done by using primer pairs *Nsi*I-f : 5′-TTTAAGGTTCCTCATGCCAAG-3′ and G_328_S-r: 5′-GCTCCAGCACTTTCATACTT-3′, and G_328_S-f: 5′-CAAGTATGAAAGTGCTGGAGC-3′(this changed E gene amino acid no. 328 from Gly to Ser; from GGT to AGT ) and *Stu*I-r 5′-CAACATGATGAGGGCTCGTA-3′ for construction of DEN-4 2A E-G_328_S; primer pairs *Nsi*I-f : 5′-TTTAAGGTTCCTCATGCCAAG-3′ and Q_438_H-r: 5′-CCAAAAACGTGGTGCACAGC-3′, and Q_438_H-f: 5′- GCTGTGCACCACGTTTTTGG-3′(this changed E gene amino acid no. 438 from Gln to His; from CAG to CAC ) and *Stu*I-r 5′-CAACATGATGAGGGCTCGTA-3′ for construction of DEN-4 2A E- Q_438_H; primer pairs *Nsi*I-f : 5′-TTTAAGGTTCCTCATGCCAAG-3′ and V_463_L-r: 5′-ATCCACAACAGTAAGAACCC-3′, and V_463_L-f: 5′-GGGTTCTTACTGTTGTGGAT-3′(this changed E gene amino acid no. 463 from Val to Leu; from GTG to CTG ) and *Stu*I-r 5′-CAACATGATGAGGGCTCGTA-3′ for construction of DEN-4 2A E- V_463_L. The second round was done by using the same primer pairs *Nsi*I-f : 5′-TTTAAGGTTCCTCATGCCAAG-3′ and *Stu*I-r 5′-CAACATGATGAGGGCTCGTA-3′. The 2,003 bp *Nsi*I - *Stu*I PCR fragments were cloned into the pJET1.2/blunt cloning vector (Fermentas Life Sciences Corp.) for amplification. A 1,412 bp region flanked by *Hpa*I and *Nsi*I restriction enzyme sites in DEN-4 2A infectious clone was replaced with *Hpa*I-*Nsi*I fragment derived from confirmed clones, which contained E-T_138_P mutation in DEN-4 2A infectious clones. To construct DEN-4 2A NS2B-G_69_R, NS2B-Q_78_H, NS2B-G_108_R, and NS2B-A_113_T infectious cDNA clones, PCR fragments containing corresponding mutations were amplified by two rounds of PCR reactions. The first round was done by using primer pairs *Stu*I-f : 5′-CACTCTTTGTGCTATCATCT-3′ and G_69_R-r: 5′-GGCTTGAGCGTGTTATGTC -3′, and G_69_R-f: 5′-GACATAACACGCTCAAGCC-3′(this changed NS2B gene amino acid no. 69 from Gly to Arg; from GGC to CGC ) and *BstB*I-r 5′- CATTATAGTTAATCTTTTCTTTC-3′ for construction of DEN-4 2A NS2B-G_69_R; primer pairs *Stu*I-f : 5′-CACTCTTTGTGCTATCATCT-3′ and Q_78_H-r: 5′-TCTTCATCATGCTTCACTTC-3′, and Q_78_H-f: 5′-GAAGTGAAGCATGATGAAGA-3′(this changed NS2B gene amino acid no. 78 from Gln to His; from CAG to CAT ) and *BstB*I-r 5′- CATTATAGTTAATCTTTTCTTTC-3′ for construction of DEN-4 2A NS2B-Q_78_H; and primer pairs *Stu*I-f : 5′-CACTCTTTGTGCTATCATCT-3′ and G_108_R -r: 5′- GGGTAGAGACGTGACACTG-3′, and G_108_R -f: 5′-CAGTGTCACGTCTCTACCC-3′(this changed NS2B gene amino acid no. 108 from Gly to Arg; from GGT to CGT ) and *BstB*I-r 5′- CATTATAGTTAATCTTTTCTTTC-3′ for construction of DEN-4 2A NS2B- G_108_R; and primer pairs *Stu*I-f : 5′-CACTCTTTGTGCTATCATCT-3′ and A_113_T-r: 5′-TGGAATTGTCAAGGGGTAG-3′, and A_113_T-f: 5′-CTACCCCTTGACAATTCCA-3′(this changed NS2B gene amino acid no. 113 from Ala to Thr; from GCA to ACA ) and emphtype

### Mouse studies

All mice were housed at the National Tsing Hua University barrier facility, and cared for according to protocols approved by the university's Institutional Animal Care and Use Committee (Permit Number: 09734). We performed each neurovirulence and in vivo hemorrhage experiment at least two times.

### Neurovirulence in suckling mice

Litters of newborn (less than 24 hrs) outbred white ICR mice (BioLASCO Taiwan Co., Ltd) were inoculated intracranially with 30 µl of mock diluent or diluent containing 10^4^ PFU of DEN-4 2A-Vero-P4, DEN-4 2A-MRC-5-P4, DEN-4 2A -Vero-P10, DEN-4 2A-MRC-5-P10, DEN-4 2AΔ30-Vero-P4, DEN-4 2AΔ30-MRC-5-P4, DEN-4 2AΔ30-Vero-P10, or DEN-4 2AΔ30-MRC-5-P10 as described previously [26], [48]. HBSS-0.4% BSA fraction V (GIBCO) was used as a diluent. Each group consisted of at least 10 newborn mice per treatment. Mice were observed for 18 days. We collected data on moribund status, paralysis, and mortality.

### Dengue virus-induced hemorrhaging mouse model

Immunocompetent C57BL/6 mice were obtained from the Jackson Laboratory (Bar Harbor, ME) and bred at the Laboratory Animal Center of the National Taiwan University College of Medicine. All mice were housed in sterile cages fitted with filtered cage tops and fed sterilized food and water. At 4–5 weeks of age, mice were intradermally inoculated with 4×10^7^ PFU DENV (in 0.4 ml of PBS) at four sites on the upper back. Control mice were given PBS and culture medium in the same manner. Mice were sacrificed 3 days post-infection. Subcutaneous tissues in the back, abdomen, and axillary areas and thorax were exposed to observe hemorrhaging.

## Supporting Information

Table S1 **Comparison of estimated error rates for DEN-4 2A and DEN-4 2AΔ30 RNA polymerase in the C-prM-E, NS2B-NS3 and NS4B-NS5 regions following Vero and MRC-5 cell passages.**
(DOC)
