# Supplementary material for: Dengue Type 4 Live-Attenuated Vaccine Viruses Passaged in Vero Cells Affect Genetic Stability and Dengue-Induced Hemorrhaging in Mice
Source: PLoS One. 2011 Oct 28;6(10):e25800. doi: 10.1371/journal.pone.0025800 (PMC3203870; doi:10.1371/journal.pone.0025800)
Supplement: Table S1 — Comparison of estimated error rates for DEN-4 2A and DEN-4 2AΔ30 RNA polymerase in the C-prM-E, NS2B-NS3 and NS4B-NS5 regions following Vero and MRC-5 cell passages. (DOC) [file pone.0025800.s001.doc]

Table S1

| **Virus** | **Cell Line** | **Number of DEN Polymerase Mistakes** | | | **DEN Polymerase Fidelity Per Copied Nucleotide** | | |
| --- | --- | --- | --- | --- | --- | --- | --- |
|  |  | **C-prM-E** | **NS2B-NS3** | **NS4B-NS5** | **C-prM-E** | **NS2B-NS3** | **NS4B-NS5** |
| **DEN-4 2A**  **infectious clone** | **Vero** | **37** | **28** | **0** | **(1.05~1.16) ×10^-6^** | **(7.98~8.81) ×10^-7^** | **0** |
|  | **MRC-5** | **2** | **0** | **0** | **(6.29~7.30) ×10^-8^** | **0** | **0** |
| **DEN-4 2AΔ30**  **infectious clone** | **Vero** | **23** | **39** | **0** | **(6.57~7.26) ×10^-7^** | **(1.11~1.23) ×10^-6^** | **0** |
|  | **MRC-5** | **0** | **0** | **0** | **0** | **0** | **0** |
